# Characteristics and outcomes of patients with pelvic organ prolapse: an analysis of data from Mulago National Referral Hospital from 2007–2016

**DOI:** 10.4314/ahs.v23i1.43

**Published:** 2023-03

**Authors:** Josaphat Byamugisha, Barageine Justus, Othman Kakaire, Nalubwama Haddy, Obore Susan, Abner Korn, David Christopher Mukasa, Mwanje Haruna, Alia Godfrey, El Ayadi Alison

**Affiliations:** 1 Department of Obstetrics and Gynecology, School of Medicine, College of Health Sciences, Makerere University, Kampala, Uganda; 2 Directorate of Obstetrics and Gynecology, Mulago National Referral Hospital, Kampala, Uganda; 3 Department of Obstetrics, Gynecology and Reproductive Sciences, UCSF, San Francisco; 4 University Hospital, Makerere University, Kampala, Uganda

**Keywords:** Pelvic organ prolapse, Mulago Hospital, Uganda

## Abstract

**Introduction:**

Pelvic organ prolapse (POP) is associated with negative physical, social, psychological, and sexual experiences. There is paucity of data in low and middle income countries like Uganda. The purpose of this study was to describe clinical characteristics and outcomes of patients undergoing surgery for POP from 2007 to 2016.

**Methods:**

The study was conducted at Mulago National Referral Hospital from 2014 to 2016. We conducted a retrospective review of the urogynecology surgical records using a standardized medical record abstraction form. Data of 222 POP patients were abstracted and managed using REDCap. Analysis was performed using Stata statistical software, v14.

**Results:**

The mean participant age and parity was 57 years and 7 respectively. Ninety four percent of participants presented with a mass protruding from the vagina, 38% with uterine prolapse and 32% with cystocoele. Anaemia and hypertension were common comorbidities. Women underwent a variety of surgery types, and 35% experienced persistent pain post-operatively. At hospital discharge, 83% had achieved either complete resolution or improvement in their condition.

**Conclusions:**

Measures encouraging presentation for care as soon as symptoms are experienced and reduction of total fertility rate will be beneficial. Patients with POP should be routinely screened for anaemia and hypertension.

## Introduction

Pelvic organ prolapse refers to displacement of the bladder, uterus, vagina, small bowel, or rectum as a result of weakened or damaged pelvic muscles and ligaments. Variability in diagnostic criteria and inclusion of both symptomatic and asymptomatic women results in reports of widely varied prevalence estimates, ranging from 3-50%^1^. Existing data from Europe suggests that high parity, age, obesity and vaginal delivery are the primary risk factors for pelvic organ prolapse^2^–^5^. Parity is cited to have the highest correlation^2^. Level of trauma to the pelvic floor and supports varies proportinally with increasing vaginal parity^6^. Ugandan women have a high Total Fertility Rate of 5.4 7. Data on genetic predisposition to pelvic organ prolapse found positive association^6^.

Pelvic organ prolapse is associated with negative physical, social, psychological, and sexual experiences^7^,^8^. Studies in Ethiopia and Uganda assessing the prevalence of depressive symptoms identified high risk among women with pelvic organ prolapse^9^, ^10^. These findings are supported by qualitative work in the United States which found poorer emotional health and subjective well-being^11^. The economic impact of pelvic organ prolapse is thought to be enormous. There is, however, inadequate data on the actual figures in terms of financial cost of treatment^12^. Similary, access to care for Ugandan patients with Pelvic organ prolapse is limited.

The demand for treatment of pelvic organ prolape in United States is anticpated to increase in tandem with the predicted 46% increase in burden, by 2050^13^.

Generally, there are two major categories of treatment options, the surgical and the conservative methods. Both surgical correction and conservative treatment using pessaries have been found to be viable alternatives among Ugandan women^14^, ^15^. Surgical treatment was found to significantly improve the quality of life in Ugandan women^15^. In Mulago National Referral Hospital, that served the bigger majority of the urban poor, all the patients with pelvic organ prolapse underwent surgical management.

The purpose of this study was to describe clinical characteristics and outcomes of patients undergoing surgery for pelvic organ prolapse from 2007 to 2016.

## Materials and methods

### Study setting

The study was conducted at Mulago National Referral and Teaching Hospital (Mulago Hospital) in Uganda, located about 3km by road from the city centre of the capital, Kampala. Also the teaching hospital for Makerere University, serves a large population within a catchment radius of up to 20km, but additionally receives referrals from all parts of the country and beyond. The hospital is one of the busiest maternity centres worldwide, and conducts approximately 33,000 deliveries annually^16^ and provides tertiary specialised gynaecological services including Urogynaecology. The hospital is run by urogynaecological surgeons that train for FIGO in fistula repairs^19^. Additional urogynecological services provided include management of ureteric injuries^20^. Our study focused on patients with pelvic organ prolapse.

### Study design

We conducted a retrospective review of the urogynecology surgical records over the period 2014-2016 using a standardized medical record abstraction form developed expressly for this purpose. The data extracted represented surgical operations that occurred between 2007-2016. Records for a total of 222 pelvic organ prolapse patients were identified and abstracted

Data abstracted included participant socio-demographic characteristics, obstetric history, presenting symptoms, type and grade of prolapse, surgery types, post-operative complications, and status at hospital discharge. Study data were collected and managed using REDCap electronic data capture tools hosted at the University of California, San Francisco17. Data were aggregated for presentation into two-year periods, and we describe patient characteristics, treament and outcomes across the study time frame.Means and standard deviations were calculated for continuous variables age and parity across each two-year period. Numbers and proportions were calculated across categorical variables, comorbidities, duration of symptoms, presenting symptoms, prolapse type and grade, surgery type, complications and status. Surgeries received are presented in combinations given. All data analysis was performed within Stata statistical analysis software, v14 (College Station, Texas).

### Ethical considerations

Approval to conduct the research was obtained from Makerere University School of Medicine Research and Ethics Committee (Ref NO: 2014-052) and the Uganda National Council for Science and Technology (Ref ADM l54/2l2/0l)

## Results

Over the period 2007-2016, data from 222 women who underwent surgical treatment for pelvic organ prolapse were abstracted from medical records (Table 1). Across this time period, mean patient age was 56.8 (SD 14.9) and mean parity was 7.4 (SD 3.0). Thirty six percent of the women had evidence of anemia and one-fifth presented with hypertension.

**Table 1:** Characteristics, treatment, and outcomes of pelvic organ prolapse patients, Mulago National Referral Hospital, 2007–2016

	2007–8		2009–10		2011–12		2013–14		2015–16		Total	
	n=15		n=45		n=35		n=82		n=45		n=222	
	N	%	N	%	N	%	N	%	n	%	n	%
Age^a^	59.3 (12.8)		59.1 (12.8)		54.8 (16.3)		54.0 (16.4)		60.0 (12.7)		56.8 (14.9)	
Para^a^	8.8 (2.9)		8.0 (2.9)		6.7 (3.3)		6.9 (3.0)		7.7 (2.7)		7.4 (3.0)	
Comorbidities												
Anemia^b^	1	20.0	21	63.6	10	34.5	22	30.1	8	23.5	62	35.6
Hypertension	4	26.7	11	24.4	5	14.3	13	15.9	12	26.7	45	20.3
Duration of Symptoms	n=15		n=41		n=35		n=81		n=39		n=211	
<1 Mo	0	0.0	0	0.0	1	2.9	2	2.5	0	0.0	3	1.4
1–6 Mo	2	13.3	8	19.5	4	11.4	18	22.2	7	18.0	39	18.5
7–12 Mo	1	6.7	11	26.8	8	22.9	18	22.2	6	18.0	45	21.3
1–2 Yrs	3	20.0	8	19.5	4	11.4	17	21.0	13	30.8	44	20.9
3+ Yrs	9	60.0	6	34.2	18	51.4	26	32.1	13	33.3	80	37.9
Presenting Symptoms												
Mass protruding per vagina	13	86.7	44	97.8	33	94.3	74	90.2	45	100.0	209	94.1
Urinary												
frequency/retention	3	20.0	9	20.0	7	20.0	10	12.2	13	28.9	42	18.9
Pelvic pain	0	0.0	12	26.7	10	28.6	3	3.7	2	4.4	27	12.2
Backache	3	20.0	4	8.9	5	14.3	3	3.7	3	6.7	18	8.1
Other symptom	1	6.7	1	2.2	3	8.6	3	3.7	6	13.3	14	6.3
Constipation	0	0.0	3	6.7	3	8.6	1	1.2	12	6.7	19	4.5
Pelvic heaviness/discomfort	1	6.7	2	4.4	1	2.9	1	1.2	3	6.7	8	3.6
Coital difficulty	0	0.0	0	0.0	0	0.0	1	1.2	0	0.0	1	0.5
Type of Prolapse	n=12		n=42		n=34		n=79		n=43		n=212	
Cystocoele	6	50.0	12	28.6	12	35.3	29	36.7	8	18.6	67	31.9
Urethrocoele	0	0.0	0	0.0	0	0.0	1	1.3	1	2.3	2	1.0
Rectocoele	2	16.7	2	4.8	1	2.9	8	10.1	0	0.0	13	6.2
Uterine prolapse	4	33.3	27	64.3	20	58.8	36	45.6	34	79.1	121	57.6
Vaginal vault	0	0.0	1	2.4	1	2.9	5	6.3	0	0.0	7	3.3
Prolapse Grade	n=14		n=41		n=30		n=72		n=40		n=197	
1	2	14.3	3	7.3	1	3.3	3	4.2	1	2.5	10	5.1
2	2	14.3	16	39.0	9	30.0	13	18.1	8	20.0	48	24.4
3	6	42.9	12	29.3	5	16.7	6	8.3	4	10.0	33	16.8
4 (procidentia)	4	28.6	5	12.2	8	26.7	23	31.9	21	52.5	61	31.0
Not graded	0	0.0	5	12.2	7	23.3	27	37.5	6	15.0	45	22.8
Operation												
Type/Combination												
AC, VH and P	1	6.7	15	33.3	9	25.7	18	22.0	18	40.0	61	27.5
AC, PC, VH and P	5	33.3	9	20.0	5	14.3	12	14.6	7	15.6	38	17.1
AC alone	4	26.7	4	8.9	4	11.4	18	22.0	4	8.9	34	15.3
VH alone	2	13.3	10	22.2	7	20.0	8	9.8	6	13.3	33	14.9
AC, PC and P	1	6.7	2	4.4	2	5.7	7	8.5	1	2.2	13	5.9
PC alone	1	6.7	1	2.2	1	2.9	8	9.8	0	0.0	11	5.0
Other combinationc	1	6.67	4	8.88	7	20	11	13.4	9	2	32	14.4
Post-operative complications												
Persistent pain	6	40.0	18	40.0	14	40.0	16	19.5	23	51.1	77	34.7
None	6	40.0	13	28.9	11	31.4	35	42.7	9	20.0	74	33.3
Bleeding	1	6.7	1	2.2	2	5.7	5	6.1	1	2.2	10	4.5
Infection	0	0.0	3	6.7	2	5.7	1	1.2	0	0.0	6	2.7
Urinary incontinence	0	0.0	1	2.2	1	2.9	3	3.6	1	2.2	6	2.7
Urine retention	0	0.0	1	2.2	1	2.9	1	1.2	0	0.0	3	1.4
Blocked catheter	0	0.0	0	0.0	0	0.0	0	0.0	1	2.2	1	0.5
Recurrence	0	0.0	0	0.0	1	2.9	0	0.0	0	0.0	1	0.5
Other	8	53.3	29	64.4	19	54.3	38	46.3	24	53.3	118	53.2
Status at Discharge												
Cured	1	6.7	6	13.3	3	8.6	15	18.3	2	4.4	27	12.2
Improved	14	93.3	39	86.7	29	82.9	59	72.0	42	93.3	183	82.4
Unimproved	0	0.0	0	0.0	0	0.0	2	2.4	0	0.0	2	0.9
No information	0	0.0	0	0.0	3	8.6	6	7.3	1	2.2	10	4.5

a mean(sd); AC= anterior colporrhaphy; VH: vaginal hysterectomy; PC: posterior colpoperinneorrphaphy; P: perinneorrphaphy; SF: suspension/fixation; C: colposuspension.

b Data for anemia were available by year 2007–8 (n=5), 2009–2010 (n=33), 2011–2012 (n=29), 2013–14 (n=73), 2015–16 (n=34).

c Other surgical combinations included those which comprised fewer than 2.5% by type across all years, and included the following: AC, P & SF (2.3%); P & SF (1.8%); P & other (1.8%); AC & P (0.9%); PC & P (0.9%); AC, C and P (0.9%); VH, P and other (0.9%); C (0.5%); AC & PC (0.5%); PC & VH (0.5%); PC, VH & P (0.5%); PC, C & P (0.5%); PC, P & other (0.5%); PC, P & other (0.5%); AC, PC, VH, C & P (0.5%); AC, PC, VH, P & SF (0.5%); AC, PC, VH, P & other (0.5%).

Ninety four percent of women presented with a mass protruding from the vagina. Other presenting symptoms were less common, and included urinary frequency or retention (19%), pelvic pain (12%), and backache (8%), among others. Duration of symptoms prior to presentation for tratment varied, with 38% reporting symptoms for three or more years, 21% percent for 1-2 years, 21% for 7-12 months and 19% for 1-6 months.

Most women were diagnosed with uterine prolapse (58%) or cystocoele (32%). Distribution across prolapse grade was grade 1 (5%), 2 (24%), 3- (17%), 4 (31%), with 23% not graded. These were described using the Baden-Walker grading system of comprising grades 1, 2, 3 and 4. Women underwent a variety of surgery types and combinations: the most common was anterior colporrhaphy, vaginal hysterectomy and perineorrphaphy (28.0%). Notably, less than three percent of these patients had a suspension/fixation. Thirty five percent of women experienced persistent pain post-operatively and thirty three percent reported no post-operative problems. Fewer women experienced bleeding (4.5%), infection (2.7%) or urinary incontinence (2.7%). At discharge, eighty two percent of women reported improvement in their condition (partial resolution of the presenting complaints at the time of discharge) and twelve percent felt they were completely cured.

## Discussion

### Population is largely post-menopausal, high parity

Only a few studies have been done about the subject of pelvic organ prolapse in Uganda. In this retrospective review of medical records data, we describe the physical and clinical characteristics of patients managed for pelvic organ prolapse. Participants in our study were largely post-menopausal and high parity. These findings are consistent with the literature which reports menopausal women and women of higher parity to be at higher risk of pelvic organ prolapse^3^,^18^,^19^. The mean patient age in our study was 56.8 years which is significantly lower than other pelvic organ prolapse studies where mean age of Asian patients was over 70 years^19^. In the United States, menopausal women were at higher risk for prolapse^20^. Ethiopia, another low to middle income country, age of onset of pelvic organ prolapse was lower at 40 years^21^ Perhaps, the difference in patient age can be attributed to the lower socioeconomic status of African women compared to their Asian population. The mean parity of our participants was 7.4. This is in tandem with recent research in Uganda that found positive correlation with grand multipairty^22^. Data from Korea, a high income setting, found positive association of pelvic organ prolapse with comparitively lower parity of 3 or more^19^. In our study, we did not document the mode of delivery, but data from the United States demonstrates that vaginal delivery, and not just high parity, carries higher risk compared to caeserean delivery^23^.

### Lengthy time to care seeking

Just over half of our study population had symptoms for more than a year prior to seeking care, while only a small minority of 1.4% sought care within a month duration. There was significant delay in seeking health within the population. A study amongst Emirati women had similar findings of just over half (54%) not seeking help for several reasons such as lack of knowledge, fear of embarassment, hope of spontaneous resolution among others^24^. Social stigma was cited as the main barrier to seeking help in an earlier study conducted in Uganda^25^, while a qualitative study amongst Dutch women points out lack of accurate information, and recommends health education of mothers^26^.

### Advanced prolapses

Nearly all patients presented with advanced prolapse; 94% presented with a mass protruding from the vagina, one-third with grade 3 prolapse and another third with grade 4. A survey done in the USA revealed a significant number of about a quarter of women who had at least one symptom pelvic floor dysfunction^27^, however, the likelihood of seeking care depends on the severity of the illness^24^. Women who do manual physical work, with lower socioeconomic status are more prone to suffering more severe pelvic organ prolapse^28^. Mulago National Referral Hospital serves a big population of the urban poor and patients usually present with late symptoms.

### High improvement (82.4%), low cure (12.2%)

The majority of our study participants reported improvement but not cure. Both surgical and conservative treatments (such as pessaries and pelvic floor training) for pelvic organ prolapse have been shown to improve quality of life^29^, ^30^. Conservative methods such as pessaries have shown success in treatment of even advanced pelvic organ prolapse such as grades 3 and 4^31^, ^32^. Although, it remains unclear which management method has comparatively higher cure rates, treatment should aim to boost patient satisfaction and improve quality of life^33^,^34^. All participants were surgically managed. Notably, less than three percent of these patients had a suspension/fixation. Studies have shown that the majority of women who have cystocele are also likely to have apical prolapse^35^–^37^ and their surgical treatment should include apical suspension. This suggests an oppoortunity for improvement in surgical treatment of pelvic organ prolapse at Mulago hospital.

In addition to the physical symptoms of prolapse itself, women with prolapse are negatively affected socially, psychologically and sexually. Poor mental health has been reported as a common sequela of prolapse. Two prior studies in Ethiopia and Uganda assessing the prevalence of symptoms of depression identified high risk among women with pelvic organ prolapse^9^, ^10^. These findings are consistent with research from the United States and elsewhere which found poorer emotional health and subjective well-being^8^,^11^. Women with prolapse report poorer sexual functioning^7^.

### Hypertension and anaemia

In this study, we found about one-third to have evidence of anemia (36%) and one-fifth presented with hypertension (20%). Hypertension has been implicated in pelvic organ prolapse^38^,^39^. Not only has anaemia been cited as a risk factor for pelvic organ prolapse among Africans^40^, it has been been associated with increased likelihood of post-operative complications following surgical treatment^41^. Level of anemia has direct correlation with severity of complications^42^. Routine screening and treatment of hypertension and anaemia is paramount in management of patients with pelvic organ prolapse.

### Strengths

Our exploratory study included data from a census of individuals treated for prolapse at a national specialized facility in a low to middle income African country.

### Limitations

One of the challenges faced was that the paper medical records were not easy to retrieve, together with different format of the notes. The other challenge was missing data in some of the files.

## Conclusions

Most of the participants presented late and were of high parity. Measures encouraging presentation for care as soon as symptoms are experienced and aimed at reduction of total fertility rate will be useful. Continuous improvement in surgical treatment of Pelvic organ prolapse at Mulago Hospital. Patients with pelvic organ prolapse should be routinely screened for anaemia and hypertension.

## Declarations

### Availability of data and materials

The datasets used and analyzed during the study are included in the supplementary information files.
